# Resveratrol plus carboxymethyl-β-glucan in children with recurrent respiratory infections: a preliminary and real-life experience

**DOI:** 10.1186/s13052-014-0093-3

**Published:** 2014-11-23

**Authors:** Alfonso Maria Varricchio, Michele Capasso, Antonio della Volpe, Luigi Malafronte, Nicola Mansi, Attilio Varricchio, Giorgio Ciprandi

**Affiliations:** Associazione Italiana Vie Aeree (AIVAS): Study Group on Respiratory Infections, Via P. Boselli 5, 16146 Genoa, Italy

**Keywords:** Resveratrol, Carboxymethyl-β-glucan, Children, Recurrent respiratory infections, Acute rhinopharyngitis

## Abstract

**Background:**

Recurrent respiratory infections (RRI), such as the presence of at least one of the following criteria: i) >6 RI per year; ii) >1 RI per month involving upper airways from September to April; iii) >3 RI involving lower airways, constitute a social problem for both their pharmaco-economic impact and the burden for the family. However, several treatment have been proposed with controversial results.

**Objective:**

As resveratrol plus carboxymethyl-β-glucan is presently available as solution for aerosol, the aim of this study was to evaluate the effects of this compound, compared to saline solution, whether it is able to prevent RRI in children.

**Design:**

The study was designed as real-life, randomized. Globally, 82 children (49 males, mean age 8.1 ± 2.6 years) with acute rhinopharyngitis and RRI were enrolled. Resveratrol plus carboxymethyl-β-glucan or saline isotonic solution was randomly (ratio 1:1) administered immediately after an anti-infective and anti-inflammatory 10-day treatment (tiamphenicol associated with acetylcisteine plus beclomethasone dipropionate) for the acute rhinopharyngitis. Investigated treatments lasted 20 days. Days with respiratory symptoms, fever, medication use, medical visits, and school absences were evaluated. Children were visited 30, 60, and 90 days after starting treatments.

**Results:**

The active compound was able to significantly reduce the number of days with nasal obstruction (p < 0.001), rhinorrhea (p < 0.001), sneezing (p < 0.001), cough (p = 0.002), fever (p < 0.001), medication use (p < 0.001), medical visits (p < 0.001), and school absence (p < 0.001).

**Conclusions:**

This preliminary and real-life study could suggest that an aerosolized solution containing resveratrol plus carboxymethyl-β-glucan might exert preventive effects in children with RRI.

## Introduction

Respiratory infections (RI) during infancy and childhood are a relevant issue. The definition of recurrent RI (RRI) is based on of the presence of at least one of the following criteria: i) >6 RI per year; ii) >1 RI per month involving upper airways from September to April; iii) >3 RI involving lower airways [1]. As many children suffer from RRI, it determines a relevant impact on pharmaco-economy and is a burden for both the family and the society. Therefore, their recurrence represents a challenge for the pediatrician.

Many factors may be involved in promoting and/or causing RRI, including age (for a relative immaturity of the immune system), early attending at nursery school, air and home pollution, passive smoking, low socio-economic level, and atopy [2]. In addition, virus infections may increase the probability of contracting frequent RI because of the high number of circulating viruses and the numerous sub-types [3] Viral infections are predominant, but bacterial super-infections may frequently appear. Consequently, antibiotic resistance is growing in recent years. Thus, there is interest in preventive treatment by doctors. In this regard, immunostimulants are widely used in the common practice. Many compounds are available, but the scientific demonstration of their efficacy is still lacking for most of them. In this regard, herbal medicine could represent a valid alternative to chemical compound both concerning efficacy and safety. Very recently, it is available a new product containing resveratrol plus carboxymethyl-β-glucan as solution for aerosol therapy.

Resveratrol (*trans*-3,4,5-trihydroxystilbene) is a natural non-flavonoid polyphenol and belongs to a subclass of stilbenes. It is found in various fruits and vegetables and abundant in grape skin, it functions as a phytoalexin (a class of vegetal antibiotics) so protecting the plant from environmental stress or infections. Resveratrol exerts anti-infective and anti-inflammatory activities [4]. The anti-inflammatory effects of resveratrol depend on the inhibition of the transcription factor NF-kB, mainly inhibiting Ik-B kinase [5]. Moreover, resveratrol inhibits viral replication [6,7]. A very recent study provided evidence that resveratrol inhibits the replication of rhinovirus (RV), the etiologic agent of common cold, on nasal epithelial cells and the RV-dependent expression of ICAM-1, that is the main RV receptor [8]. Resveratrol has also demonstrated anti-inflammatory and anti-asthmatic effects in mouse model of allergic asthma, diminishing IL-4 and IL-5 in plasma and bronchoalveolar lavage fluid, and suppressing bronchial hyperreactivity, lung eosinophilia, and mucus hypersecretion [9].

On the other hand, β-glucan is a polysaccharide with well-known immune-modulatory properties, including stimulation of phagocytosis by professional phagocytes, direct activation of NK cells and cytokine release [10,11]. Previously, it has been reported that resveratrol combined with β-glucan exerted relevant *in vitro* synergistic effects on immune system [12]. However, there are several differences between various sources of β-glucans regarding their clinical efficacy and mechanisms of action and that the purity of the products is strongly connected with the final clinical effect.

Very recently, it has been reported that resveratrol plus carboxymethyl-β-glucan nasal spray was able to reduce symptoms severity and antihistamine use in children with allergic rhinitis due to Parietaria sensitization [13].

However, no clinical study evaluated the capacity of this combined product in children with RRI. Therefore, the present real-life study aimed at investigating whether an aerosol therapy using a solution containing resveratrol plus carboxymethyl-β-glucan is able to prevent RRI in children.

## Materials and methods

### Population and eligibility criteria

Globally, 82 children (49 males, mean age 8.1 ± 2.6 years) with RRI were enrolled in the study. Inclusion criteria were: i) being outpatient of both gender, ii) aging 3–12 years, iii) positive history for RRI in the last 12 months (≥6 episodes), iv) acute rhinopharyngitis to be treated, and v) written informed consent of parents. Exclusion criteria were: primary or acquired immunodeficiency, clinically relevant passive smoking, previous (last 3 months) or current administration of drugs able to interfere with the study (eg, immunomodulants, homeopathic therapy, or systemic corticosteroids for at least 2 consecutive weeks), history of chronic disease (including cystic fibrosis and perennial allergy), cancer, or congenital malformation of the airways.

### Study design

A randomized, and open study was designed. Children with acute rhinopharyngitis were initially visited by primary care pediatricians who sent them to otolaryngologists for the diagnosis. Children were treated with a 10-day course of an aerosolized combined anti-infective and anti-inflammatory therapy [14]. The treatment regimen consisted of tiamphenicol 250 mg associated with acetylcisteine (Fluimucil Antibiotico, Zambon, Milan, Italy; 1/2 vial in the morning and ½ vial in the evening) plus beclomethasone dipropionate 800 mg (Clenil A, Chiesi, Parma, Italy; 1/2 vial in the morning and ½ vial in the evening). Both medications were diluted in 5 mL of saline solution. The treatment was administered through the nasal device Rinowash (AirLiquide SpA, Bovezzo, Italy) connected to an aerosol nebulizer with pneumatic compressor (1.5 bar per 5 L/min) (Moby-neb, AirLiquide SpA, Bovezzo, Italy). At the end of this treatment, children were randomly (ratio 1.1) assigned to two groups: Group A as control, receiving saline solution, and Group B as actively-treated.

The outcomes were: the number of days with nasal obstruction, rhinorrhea, sneezing, cough, fever, medication use (including anti-inflammatory, fever-reducer, and antibiotic), the number of medical visits, and the number of days of school absence.

This study was conducted on private patients on the basis of the common clinical practice and according to good clinical practice study guidelines in compliance with the Helsinki Declaration.

### Study treatment

At the end of the anti-infective and anti-inflammatory treatment, children were randomly assigned to two groups: Group A as control, receiving saline isotonic solution 5 mL, and Group B as actively-treated with resveratrol plus carboxymethyl-β-glucan (Linfovir Plus nasal drops, Noos, Rome, Italy) 12 drops diluted in saline isotonic solution 4 mL, both of them b.i.d. for 20 days. Both treatments were administered by Rinowash device.

### Safety

Safety and tolerability were evaluated on the basis of the number and type of adverse events recorded according to the rules of good clinical practice.

### Study procedures

The investigators diagnosed RI on the basis of the symptoms reported by the parent, as previously defined [14]. The RI diagnosis was made when at least 2 symptoms or fever (axillary temperature ≥38°C), in addition to one other symptom, were present for at least 48 hours. The symptoms taken into consideration for this diagnostic purpose were: mucopurulent rhinorrhea, stuffy or dripping nose or both, sore-throat, cough (dry or productive), otalgia (earache), fever, dyspnea, and mucupurulent secretion. RRI diagnosis was performed on history, such as patient’s recall of symptoms.

The children were examined at study entry, and 30 days (T1), 60 (T2), and 90 (T3) after starting the investigated treatments. The study started in November 2013 and ended in March 2014.

At each visit, history of infections occurring during the preceding period was taken. Clinical examination was also performed. All assessed parameters were regularly recorded on a daily diary card.

### Statistical analysis

The sample size was calculated by log-rank test with power at 90% and α error at 5%: 35 subjects per arm were considered sufficient based a supposed difference in efficacy of at least 30%. Randomization was performed per blocks following the Wichmann-Hill model.

Quantitative parameters were reported as means and standard deviations (SD). The test was used as a nonparametric counterpart. For the intra-group analysis, data were analyzed using the Anova Friedman test and thereafter a post-hoc tests in order to decide which groups are significantly different from each other. Comparison of quantitative data between the two groups of patients was made by the U-Mann–Whitney test.

The mean with standard error (SE) was used in graphs. All statistical tests were two sided; a P value of less than 0.05 was considered as statistically significant. A statistical software program (StatSoft Italia s.r.l. 2005. Statistica 7.1) was used for all the analyses.

## Results

Randomly (ratio 1:1), children were subdivided into two groups: A (treated with saline solution), including 40 children and B (treated with resveratrol plus carboxymethyl-β-glucan), including 42 children. Both groups were homogeneous considering the mean age (8.1 ± 2.4 years for Group A and 8.2 ± 1.9 years for Group B), the age classes (respectively 89% and 84% of children belonged to the 6–10 years class), number of previous RI (respectively 6.5 and 6.8 RI in the past year; most of them were upper airways infections) All children completed the study. Both treatments were well tolerated and safe as no relevant adverse events occurred.

Clinical data are reported for both groups in Table 1.

**Table 1:** **Clinical symptoms (days with symptoms) evaluated in Group A and in Group B at T1, T2, and T3**

**Symptoms**	**Group A**			**P value**	**Group B**			**P value**
	**T1**	**T2**	**T3**		**T1**	**T2**	**T3**	
**Nasal obstruction**	**3.3 ± 1.7**	**6 ± 4.4**	**8.3 ± 5.2**	**< 0.001**	**3.7 ± 1.7**	**1.2 ± 1.4**	**1.6 ± 2.2**	**< 0.001**
**Rhinorrhea**	**5 ± 1.5**	**8.5 ± 6.1**	**12.1 ± 5.7**	**< 0.001**	**4.7 ± 1.6**	**3 ± 2.9**	**2.8 ± 3**	**< 0.001**
**Sneezing**	**0.7 ± 1**	**1.6 ± 2.3**	**1.3 ± 1.7**	**< 0.001**	**0.8 ± 1.3**	**0.3 ± 1.1**	**0.3 ± 1.1**	**< 0.001**
**Cough**	**3.5 ± 3.3**	**3.5 ± 3**	**3.6 ± 3.2**	**N.S.**	**3.5 ± 3.2**	**1.9 ± 2.7**	**2.2 ± 3.1**	**0.002**
**Fever**	**1.5 ± 1.7**	**1.2 ± 2.5**	**1.2 ± 1.5**	**N.S.**	**0.3 ± 0.7**	**1.8 ± 2.4**	**0.2 ± 0.5**	**<0.001**
**Medications**	**9.3 ± 0.7**	**5.5 ± 3**	**5.9 ± 2.8**	**<0.05**	**9.1 ± 0.6**	**1.3 ± 1.8**	**2.5 ± 2.9**	**<0.001**
**Medical visits**	**0.5 ± 0.7**	**1.5 ± 1**	**1.5 ± 1.2**	**<0.001**	**0.4 ± 0.6**	**0.6 ± 0.9**	**0.8 ± 1.4**	**<0.001**
**Absences at school**	**1.4 ± 2**	**2.3 ± 2.1**	**2.3 ± 2**	**<0.005**	**0.4 ± 0.7**	**1 ± 1**	**1.3 ± 1.8**	**<0.001**

### Intragroup analysis

#### Group A

**Figure 1:**
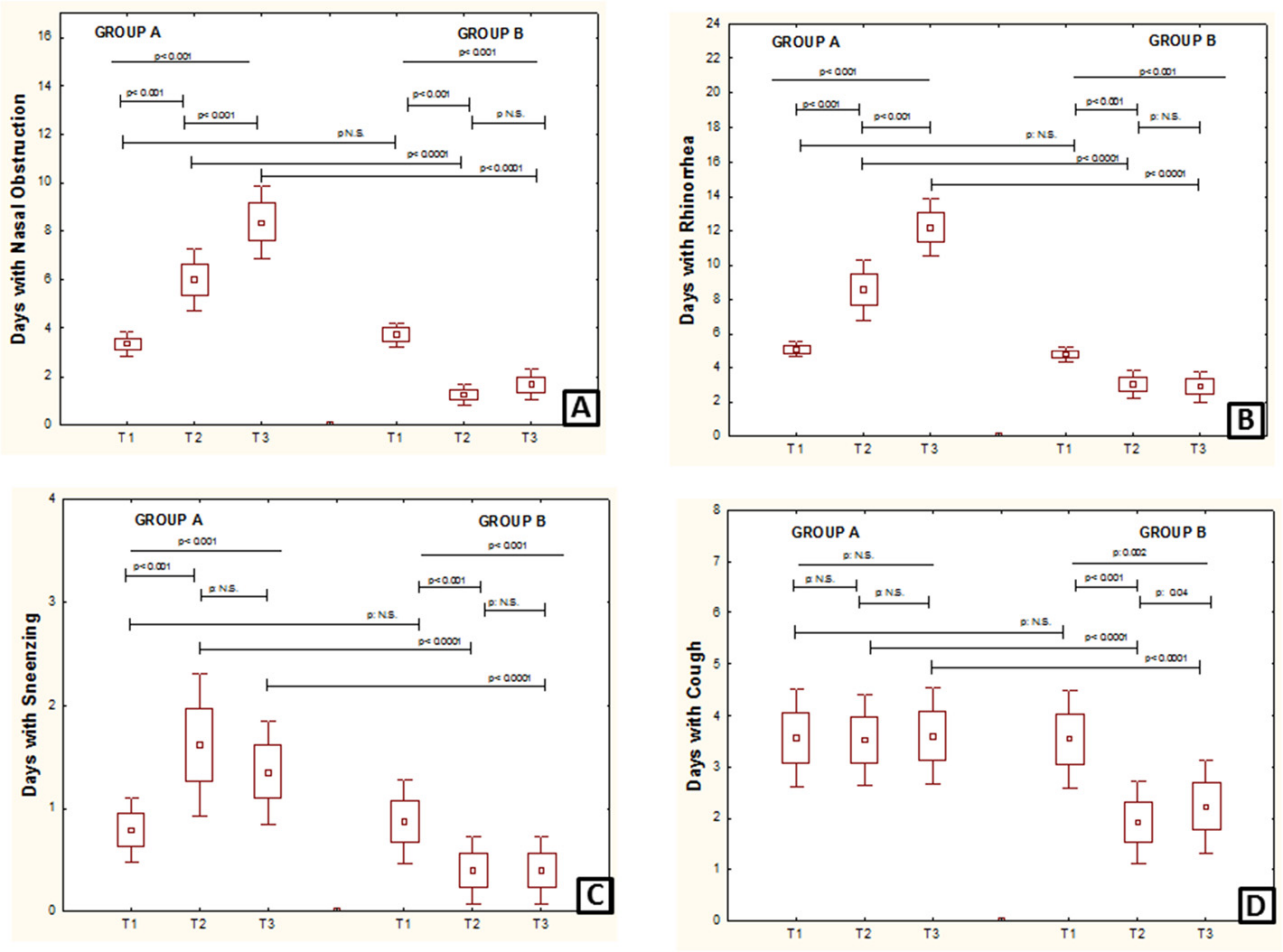
**Clinical data in control (Group A) and actively-treated (Group B) patients. A** = number of days with nasal obstruction; **B** = number of days with rhinorrhea; **C** = number of days with sneezing; **D** = number of days with cough in Group A (control) and Group B (resveratrol plus carboxymethyl-β-glucan) assessed 30 days (T1), 60 days (T2), and 90 days (T3) after anti-infective and anti-inflammatory treatment.

The number of days with nasal obstruction progressively increased: 3.3 + 1.7 at T1; 6 + 4.4 at T2; 8.3 + 5.2 at T3 (p < 0.001), as shown in Figure 1A. The number of days with rhinorrhea progressively increased: 5 + 1.5 at T1; 8.5 + 6.1 at T2; 12.1 + 5.7 at T3 (p < 0.001), as shown in Figure 1B. The number of days with sneezing progressively increased: 0.7 + 1 at T1; 1.6 + 2.3 at T2; 1.3 + 1.7 at T3 (p < 0.001), as shown in Figure 1C. The number of days with cough did not change: 3.5 + 3.3 at T1; 3.5 + 3 at T2; 3.6 + 3.2 at T3 (p = n.s.), as shown in Figure 1D.

**Figure 2:**
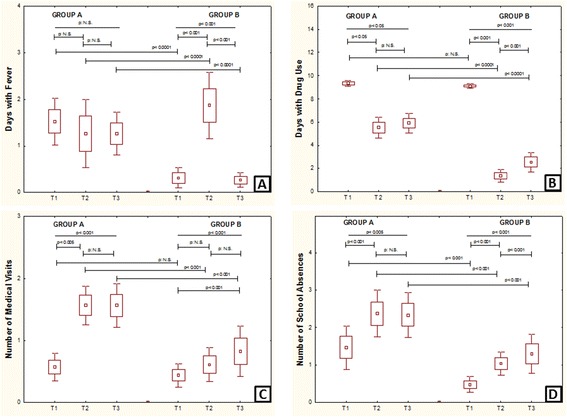
**Clinical and pharmaco-economic data in control (Group A) and actively-treated (Group B) patients. A** = number of days with fever; **B** = number of days with medication use; **C** = number ofmedical visits; **D** = number of days with school absence in Group A (control) and Group B (resveratrol plus carboxymethyl-β-glucan) assessed 30 days (T1), 60 days (T2), and 90 days (T3) after anti-infective and anti-inflammatory treatment.

The number of days with fever remained unchanged: 1.5 + 1.7 at T1; 1.2 + 2.5 at T2; 1.2 + 1.5 at T3 (p = n.s.), as shown in Figure 2A. The number of days with use of any medication progressively decreased: 9.3 + 0.7 at T1; 5.5 + 3 at T2; 5.9 + 2.8 at T3 (p < 0.05), as shown in Figure 2B. The number of medical visits increased: 0.5 + 0.7 at T1; 1.5 + 1 at T2; 1.5 + 1.2 at T3 (p < 0.001), as shown in Figure 2C. The number of days with school absence increased: 1.4 + 2 at T1; 2.3 + 2.1 at T2; 2.3 + 2 at T3 (p < 0.005), as shown in Figure 2D.

#### Group B

The number of days with nasal obstruction progressively decreased: 3.7 + 1.7 at T1; 1.2 + 1.4 at T2; 1.6 + 2.2 at T3 (p < 0.001), as shown in Figure 1A. The number of days with rhinorrhea progressively decreased: 4.7 + 1.6 at T1; 3 + 2.9 at T2; 2.8 + 3 at T3 (p < 0.001), as shown in Figure 1B. The number of days with sneezing progressively decreased: 0.8 + 1.3 at T1; 0.3 + 1.1 at T2; 0.3 + 1.1 at T3 (p < 0.001), as shown in Figure 1C. The number of days with cough decreased 3.5 + 3.2 at T1; 1.9 + 2.7 at T2; 2.2 + 3.1 at T3 (p = 0.002), as shown in Figure 1D.

The number of days with fever decreased: 0.3 + 0.7 at T1; 1.8 + 2.4 at T2; 0.2 + 0.5 at T3 (p < 0.001), as shown in Figure 2A. The number of days with use of any medication decreased: 9.1 + 0.6 at T1; 1.3 + 1.8 at T2; 2.5 + 2.9 at T3 (p < 0.001), as shown in Figure 2B. The number of medical visits progressively increased: 0.4 + 0.6 at T1; 0.6 + 0.9 at T2; 0.8 + 1.4 at T3 (p < 0.001), as shown in Figure 2C. The number of days with school absence increased: 0.4 + 0.7 at T1; 1 + 1 at T2; 1.3 + 1.8 at T3 (p < 0.001), as shown in Figure 2D.

### Intergroup analysis

The days with nasal obstruction were significantly less in Group B at T2 and T3 (p < 0.0001 for both), reported in Figure 1A. The days with rhinorrhea were significantly less in Group B at T2 and T3 (p < 0.0001 for both), reported in Figure 1B. The days with nasal sneezing were significantly less in Group B at T2 and T3 (p < 0.0001 for both), reported in Figure 1C. The days with cough were significantly less in Group B at T2 and T3 (p < 0.0001 for both), reported in Figure 1D.

The days with fever were significantly less in Group B at T1, T2 and T3 (p < 0.0001 for all), reported in Figure 2A. The days with any medication use were significantly less in Group B at T2 and T3 (p < 0.0001 for both), reported in Figure 2B. The number of days with visits were significantly less in Group B at T2 and T3 (p < 0.001 for both), reported in Figure 2C. The number of days with school absence was significantly less in Group B at T1, T2 and T3 (p < 0.001 for all, reported in Figure 2D).

## Discussion

Recurrent respiratory infections are a challenging problem in childhood. Thus, RRI prevention should be a demanding objective. The present real-life study aimed at investigating whether resveratrol plus carboxymethyl-β-glucan nasal solution can be able to prevent RRI in children.

Actually, this therapy was able to affect RRI outcomes. Nasal symptoms trend to increase in children treated with the saline solution alone. It is interesting to note that the control children progressively worsened symptoms over the time. On the contrary, children treated with resveratrol plus carboxymethyl-β-glucan had progressively less days with nasal symptoms. Cough and fever remained unchanged in control group, whereas the number of days with cough and fever was significantly reduced by the active treatment. Medication use was similar in both groups during the first month after the anti-infective treatment, but further actively-treated children experienced less medication use. Medical visits increased over the time since the anti-infective treatment, but the increase was significantly lower in children treated with resveratrol plus carboxymethyl-β-glucan. Finally, the trend of school absences was significantly reduced by resveratrol plus carboxymethyl-β-glucan.

This real-life experience provides some interesting data that seem to be interesting from a clinical, pharmaco-economic, and social point of view. Resveratrol plus carboxymethyl-β-glucan is able to significantly reduce nasal symptoms, cough, and fever. In addition, resveratrol plus carboxymethyl-β-glucan reduce the medication use and the medical visits: both aspects may have direct and indirect impact on financial resources. The reduction of school absence may be particularly important for parents, often obliged to be absent from work to cure their children. On the other hand, it has to be considered that also saline solution could exert a preventive effect on RRI, as nasal lavage may be able of removing microbes, inflammatory cells and mediators. In this regard, it has been very recently demonstrated that thermal water was able of reducing the recurrence of R.I. in children with RRI [15]. Moreover, a recent double-blind, placebo-controlled, randomised, multicentre study investigated a group of children with RRI [16]. Children were randomised into: i) an active group, treated with Imunoglukan P4H® syrup (with pleuran-β-glucan from Pleurotus ostreatus and vitamin C), or ii) a placebo group (vitamin C only). Findings showed the 36% of the actively-treated children did not suffer from any respiratory infections throughout the treatment, compared to 21% in the placebo group (p < 0.05). Imunoglukan P4H® significantly decreased the frequency of flu and flu-like disease. the number of lower respiratory tract infections, as well as significantly modulated humoral and cellular immunity.

Therefore, this preliminary study might suggest that resveratrol plus carboxymethyl-β-glucan could be an interesting option in children with RRI to prolong the efficacy of anti-infective and anti-inflammatory treatment and to prevent RI recurrence.

On the other hand, the main shortcomings of this study are: to be an open study, the lack of objective assessment of symptoms, mainly concerning endoscopic assessment, and the limited number of treated patients. For these reasons, further rigorous studies should be designed to answer to these issues.

In conclusion, the present preliminary study could suggest that a solution containing resveratrol plus carboxymethyl-β-glucan, aerosolized by rhinowash, might prevent the RRI in children with RRI after an anti-infective and anti-inflammatory standard therapy.

## References

[1] Gruppo di Studio di Immunologia della Società Italiana di Pediatria (1988). Le infezioni ricorrenti nel bambino: definizione ed approccio diagnostico. Riv Immunol Allergol Ped.

[2] Cazzola M, Anaupurapu S, Page CP (2011). Polyvalent mechanical bacterial lysate for the prevention of recurrent respiratory infections: a meta-analysis. Pulm Pharmacol Ther.

[3] Griffin MR, Walker FJ, Iwane MK, Weinberg GA, Staat MA, Erdman DD (2004). New vaccine surveillance metwork study group: epidemiology of respiratory infections in young children: insights from the new vaccine surveillance network. Pediatr Infect Dis J.

[4] Bishayee A, Waghray A, Barnes KF, Mbimba T, Bhatia D, Chattterjee M, Darvesh AS (2010). Suppression of the inflammatory cascade is implicated in resveratrol chemoprevention of experimental hepatocarcinogenesis. Pharm Res.

[5] Holmes-Mcnary M, Baldwin AS (2000). Chemopreventive properties of trans-resveratrol are associated with inhibition of activation of the ikappab kinase. Cancer Res.

[6] Zang N, Xie X, Deng Y, Wu S, Wang L, Peng C, Li S, Ni K, Luo Y, Liu E (2011). Resveratrol-mediated gamma interferon reduction prevents airway inflammation and airway hyperesponsiveness in respiratory syncytial virus-infected immunocompromised mice. J Virology.

[7] Palamara AT, Nencioni L, Aquilano K, De Chiara G, Hernandez L, Cozzolino F, Ciriolo MR, Garaci E (2005). Inhibition of influenza a virus replication by resveratrol. J Infect Dis.

[8] Nardis C, Mattia E, De Leo A, Francioso A, Mosca L, Mastromarino P (2013). Resveratrol inhibition of human rhinovirus replication. Virologie.

[9] Lee M, Kim S, Kwon O, Oh S, Lee H, Ahn K (2009). Anti-inflammatory and anti-asthmatic effects of resveratrol, a polyphenolic stilbene, in a mouse model of allergic asthma. Int Immunoparmacology.

[10] Cleary JA, Kelly GE, Husband AJ (1999). The effect of molecular weight and β-1,6-linkages on priming of macrophage function in mice by (1,3)-β-D-glucan. Immunol Cell Biol.

[11] Vetvicka V, Volny T, Saraswat-Ohria S (2007). Glucan and resveratrol complex – possible synergistic effects on immune system. Biomed Pap Med Fac Univ Palacky Olomouc Czech Repub.

[12] Vetvicka V, Vetvickova J (2012). Combination of glucan, resveratrol and vitamin c demonstrates strong anti-tumor potential. Anticancer Res.

[13] Miraglia del Giudice M, Maiello N, Capristo C, Alterio E, Capasso M, Perrone L, Ciprandi G (2014). Resveratrol plus carbossimetyl-β-glucan reduces nasal symptoms in children with pollen-induced allergic rhinitis. Curr Med Res Op.

[14] Varricchio A, Capasso M, Di Gioacchino M, Ciprandi G (2008). Inhaled thiamphenicol and acetilcysteine in children with acute bacterial rhinopharyngitis. Int J Immunopathol Pharm.

[15] Varricchio A, Giuliano M, Capasso M, Del Gaizo D, Ascione E, De Lucia A, Avvisati F, Capuano F, De Rosa G, Di Mauro F, Ciprandi G (2013). Salso-sulphide thermal water in the prevention of recurrent respiratory infections in children. Int J Immunopathol Pharmacol.

[16] Jesenak M, Majtan J, Rennerova Z, Kyselovic J, Banovcin P, Hrubisko M (2013). Immunomodulatory effect of pleuran (β-glucan from Pleurotus ostreatus) in children with recurrent respiratory tract infections. Int Immunopharmacol.

